# CD11c-CD8 Spatial Cross Presentation: A Novel Approach to Link Immune Surveillance and Patient Survival in Soft Tissue Sarcoma

**DOI:** 10.3390/cancers13051175

**Published:** 2021-03-09

**Authors:** Yanhong Su, Panagiotis Tsagkozis, Andri Papakonstantinou, Nicholas P. Tobin, Okan Gultekin, Anna Malmerfelt, Katrine Ingelshed, Shi Yong Neo, Johanna Lundquist, Wiem Chaabane, Maya H. Nisancioglu, Lina W. Leiss, Arne Östman, Jonas Bergh, Saikiran Sedimbi, Kaisa Lehti, Andreas Lundqvist, Christina L. Stragliotto, Felix Haglund, Monika Ehnman

**Affiliations:** 1 Department of Oncology-Pathology, Karolinska Institutet, BioClinicum J6:20, Visionsgatan 4, 171 64 Solna, Sweden; yanhong.su@ki.se (Y.S.); andri.papakonstantinou@sll.se (A.P.); nick.tobin@ki.se (N.P.T.); anna.malmerfelt@ki.se (A.M.); shiyong.neo@ki.se (S.Y.N.); johanna.lundquist@stud.ki.se (J.L.); wiem.sivler@ki.se (W.C.); maya.nisancioglu@ki.se (M.H.N.); lina.wik.leiss@ki.se (L.W.L.); Arne.Ostman@ki.se (A.Ö.); jonas.bergh@ki.se (J.B.); Andreas.Lundqvist@ki.se (A.L.); Felix.Haglund@ki.se (F.H.); 2 Department of Molecular Medicine and Surgery, Karolinska Institutet and Muskuloskeletal Tumor Service, Karolinska University Hospital, 171 76 Stockholm, Sweden; panagiotis.tsagkozis@sll.se; 3 Department of Breast Cancer, Endocrine Tumors and Sarcoma, Karolinska University Hospital, 171 76 Stockholm, Sweden; christina.linder-stragliotto@sll.se; 4 Department of Microbiology, Tumor and Cell Biology, Karolinska Institutet, 171 65 Solna, Sweden; okan.gultekin@ki.se (O.G.); katrine.ingelshed@ki.se (K.I.); saikiran.sedimbi@ki.se (S.S.); kaisa.lehti@ki.se (K.L.); 5 Centre for Cancer Biomarkers CCBIO, University of Bergen, 5021 Bergen, Norway; 6 Department of Biomedical Laboratory Science, Norwegian University of Science and Technology, 7491 Trondheim, Norway; 7 Department of Clinical Pathology and Cytology, Karolinska University Hospital, 171 76 Stockholm, Sweden

**Keywords:** soft tissue sarcoma, UPS, liposarcoma, tumor microenvironment, immune cells, CD11c, CD8, cross presentation, antigen-presenting cells

## Abstract

**Simple Summary:**

Immune cells can be powerful regulators of tumor growth and disease progression. Yet, the potential role of professional antigen-presenting cells in soft tissue sarcoma is poorly explored. Both dendritic cells and macrophages may present exogenous antigens through major histocompatibility complex (MHC) class I molecules to CD8+ T cells, a process referred to as cross presentation. With the concept of cellular cross presentation in mind, the present study supports the hypothesis that CD11c+ cells in direct cell-cell contact with CD8+ T cells within the primary tumor are associated with an active anti-tumor immune microenvironment and favorable prognosis. Our work hereby defines a novel biomarker for immune surveillance where presence of spatial cross presentation at tissue level resolution is significantly associated with overall survival. Importantly, this biomarker is independent from established prognostic markers like tumor grade. Biomarkers linked to immune surveillance are expected to gain increasing attention with the advent of immunotherapy in clinical practice.

**Abstract:**

Checkpoint inhibitors are slowly being introduced in the care of specific sarcoma subtypes such as undifferentiated pleomorphic sarcoma, alveolar soft part sarcoma, and angiosarcoma even though formal indication is lacking. Proper biomarkers to unravel potential immune reactivity in the tumor microenvironment are therefore expected to be highly warranted. In this study, intratumoral spatial cross presentation was investigated as a novel concept where immune cell composition in the tumor microenvironment was suggested to act as a proxy for immune surveillance. Double immunohistochemistry revealed a prognostic role of direct spatial interactions between CD11c+ antigen-presenting cells (APCs) and CD8+ cells in contrast to each marker alone in a soft tissue sarcoma (STS) cohort of 177 patients from the Karolinska University Hospital (MFS *p* = 0.048, OS *p* = 0.025). The survival benefit was verified in multivariable analysis (MFS *p* = 0.012, OS *p* = 0.004). Transcriptomics performed in the TCGA sarcoma cohort confirmed the prognostic value of combining CD11c with CD8 (259 patients, *p* = 0.005), irrespective of *FOXP3* levels and in a *CD274* (PD-LI)-rich tumor microenvironment. Altogether, this study presents a histopathological approach to link immune surveillance and patient survival in STS. Notably, spatial cross presentation as a prognostic marker is distinct from therapy response-predictive biomarkers such as immune checkpoint molecules of the PD-L1/PD1 pathway.

## 1. Introduction

Soft tissue sarcoma (STS) is a rare, heterogeneous group of extraosseous connective tissue malignancies of embryonic mesodermal origin. Mortality rates are typically high, but the five-year overall survival depends on many tumor- and host-related factors [1,2]. Tumor spread is assessed by grouping or staging systems such as the American Joint Commission on Cancer (AJCC) staging system for sarcoma, which also contains information about tumor grade [3,4]. Grading is often performed according to the French Fédération Nationale des Centers de Lutte Contre le Cancer (FNCLCC) system, where differentiation, mitotic count and tumor necrosis together determine the grade to one (low grade), two, or three (high grade) [5,6].

During the last 20 years, numerous molecular biomarkers have been explored in STS [7,8]. These include pan-cancer proliferation markers, such as Ki-67, but also sarcoma-specific fusion genes. Some of these markers are today used in the diagnostic or response-predictive setting. Presence of PAX3-FOXO1 is for instance associated with the alveolar subtype of rhabdomyosarcoma [9], whereas the pathognomonic COL1A1-PDGFB fusion product predicts response to imatinib in advanced dermatofibrosarcoma protuberans [10]. Imatinib is also used to successfully inhibit abnormal kinase signaling in c-kit-mutated gastrointestinal stromal tumors [11].

Other molecular pathways of interest in STS are those related to cancer immunotherapy. The checkpoint inhibitor pembrolizumab, which targets the PD-L1/PD1 pathway, is approved by the U.S. Food and Drug Administration (FDA) for treatment of solid tumors with high mutational burden or high microsatellite instability if no satisfactory alternative treatment options exist. However, compared to many other tumor types, STSs often display relatively few mutations, but many copy-number variations [12]. High microsatellite instability was reported in only 0.78% of all the sarcomas included in The Cancer Genome Atlas Sarcoma (TCGA-SARC) collection [13]. Therefore, to what extent the different sarcoma subtypes will respond to immunotherapy needs to be unraveled.

Two of the most common STS subtypes where the immune microenvironment has been explored are liposarcoma and undifferentiated pleomorphic sarcoma (UPS) [12]. Liposarcomas are locally aggressive tumors and the most common sarcoma of the retroperitoneum. They are genetically characterized by ring chromosomes and/or giant marker chromosomes with amplification of the MDM2/CDK4 locus. Dedifferentiation is seen in approximately ten percent of the tumors, and is defined by a transition to a non-lipogenic high-grade sarcoma with variable histological features. The major treatment is surgical since the value of systemic treatment remains ambiguous. Novel treatment strategies for metastatic disease are highly warranted.

UPS is one of the emerging STS subtypes where immune checkpoint inhibitors could be considered beyond first line therapy. It is the most common sarcoma appearing in late adult life, and accounts for about ten percent of all STSs [14]. UPS usually appears in the extremities followed by the trunk and retroperitoneum [14]. The malignant cells tend to appear fibroblastic or myofibroblastic; however, they should not show a more specific line of differentiation [15]. Surgery is the cornerstone of non-metastatic management. Although the role of (neo)adjuvant chemotherapy and radiotherapy is still debated, patients with high-risk tumors are always considered for additional therapy pre- or postoperatively. When the disease is spread, the diagnosis is dismal, and there is an unmet need for new treatment modalities.

One of the main obstacles in STS is tumor heterogeneity. Still, there are several indications for consistent prognostic roles of tumor-infiltrating lymphocytes in the sarcoma tumor microenvironment (TME) [16,17,18]. Our recent study highlighted intratumoral B cells as being prognostic based on both immunohistochemistry (IHC) for CD20 (protein), and by gene expression analysis of the corresponding gene, *MS4A1* [19]. In contrast, CD20 expression in the peritumoral capsule, not the tumor itself, was suggested as a negative prognostic indicator [20]. To what extent an immunosuppressive TME, or the presence of tumor-associated tertiary lymphoid structures (TA-TLS), regulates lymphocyte activity remains to be established [21,22].

TA-TLSs are lymph node-like structures that have been described in human tumors of different origins and are believed to facilitate immune cell interactions, antigen presentation, and lymphocyte maturation [23,24]. Both dendritic cells and macrophages can, under the right circumstances, effectively present or cross present antigens, and thereby induce lymphocyte activation [25,26,27]. Cross presentation refers to a specific process where exogenous antigens are presented by antigen-presenting cells (APCs) through major histocompatibility complex (MHC) class I molecules to CD8+ T cells [28]. In contrast, MHC class II molecules, which are only expressed by professional APCs, mediate a CD4+ T cell response [29].

Conventional dendritic cells are known as efficient APCs in cross presentation and T cell activation, but also CD169+ macrophages that express CD11c may play a role in the presentation of dead cell-associated antigens [30,31,32]. Functional antigen presentation is, however, a highly dynamic process where also costimulatory molecules are essential. Of note is also that marker-defined immune cells are often not the same in mouse and man, and it has not yet been recognized to what extent different APC subpopulations are involved in cross presentation in STS. Subsets of myeloid cells can in addition trap tumor-infiltrating lymphocytes in long-lived interactions on the tumor margin without supporting full activation [33]. Consequently, even though the tumor immune microenvironment is essential to understand at the molecular level, it is yet only sparsely described in STS [30,34,35,36].

The present study explores the hypothesis that CD11c+ APCs in direct cell–cell contact with CD8+ T cells at the tumor site are associated with an active anti-tumor immune microenvironment and favorable prognosis. Altogether, the results demonstrated that the prognostic value of CD11c combined with CD8 is detected by independent methodologies and maintained in principally different TMEs.

## 2. Materials and Methods

### 2.1. Patient Inclusion and Follow Up

The Karolinska STS cohort (Table 1) contained 177 patients who were diagnosed through a standardized multidisciplinary approach at the Sarcoma Center Karolinska, Karolinska University Hospital [2]. Sample collection for a subset of the included patients has been described previously [19]. Additional sample material was included during the years 2015–2018 (retroperitoneal liposarcoma) and 2015–2020 (UPS). The liposarcomas and UPSs were identified through the digital records of the pathology unit and all available cases during the indicated time periods were included (consecutive inclusion). Clinical data were reviewed and cases with neo-adjuvant treatment were excluded. Surgery was the first-line treatment.

The primary UPS tumors were located in the extremities (72), trunk (31), intraabdominal (6), retroperitoneal (3), or in the head-and-neck region (3). Among patients with UPS, 13 patients received adjuvant chemotherapy (Adriamycin and Ifosfamide) and 14 patients received local post-operative radiotherapy. A subset of patients was treated according to the SSGXX protocol with radiotherapy between chemotherapy cycle 2 and 3.

The majority of liposarcomas originated from the retroperitoneum (42); other sites included trunk (3), extremities (2), and head-and-neck region (1). Among the liposarcomas, three patients received local post-operative radiotherapy, two adjuvant chemotherapy, one combination of post-operative radiotherapy and adjuvant chemotherapy, and one adjuvant tyrosine kinase inhibitor.

Patient surveillance followed existing guidelines for high-grade STS [2]. Clinical examination and chest X-ray or CT scan were done every 3 months for the first 2 years of follow-up, then bi-annually. Local recurrence and lung metastases were documented. Personal data was pseudonymized according to the General Data Protection Regulation (GDPR). Also, see patient inclusion for flow cytometry below.

The TCGA sarcoma cohort contained in total 259 cases available for analysis [37]. The dominating subtypes were leiomyosarcoma, liposarcoma, and UPS, but similar to the Karolinska STS cohort, a small number of myxofibrosarcoma, synovial sarcoma, and malignant peripheral nerve sheath tumor were included. For TCGA data assembly, normalized, batch corrected, RNA-sequencing data were accessed from NIH genomic data commons (GDC) database (https://gdc.cancer.gov; accessed date 17 August 2020) along with matching patient/tumor clinico-pathological characteristics. Neoadjuvant radiotherapy and chemotherapy were previously described as exclusion criteria [12].

### 2.2. Flow Cytometry and Cell Sorting

One of the UPS cases with confirmed CD11c–CD8 cellular interactions was available for marker coexpression analysis of CD8 and CD3 in a fresh tumor sample obtained during surgical removal of the primary tumor. Two additional cases (1 UPS and 1 liposarcoma) were available for flow cytometry only, and were consequently not included in the Karolinska STS cohort or analyzed for CD11c–CD8 interactions. There were no specific selection criteria beyond availability for the samples analyzed by flow cytometry.

Sample preparations followed our previously developed protocol [38]. Tumor resections were processed using a Tumor Dissociation Kit (Miltenyi Biotec), and tumor cells were isolated with a negative selection–based Tumor Cell Isolation Kit (Miltenyi Biotec) according to the manufacturer’s protocol. For surface marker phenotyping by fluorescence-activated cell sorting (FACS), cell suspensions were washed with FACS buffer (PBS with 5% FBS) and stained for flow cytometry after RBC lysis. Cells were incubated with fluorescence-conjugated antibodies for 20 min at 4 °C (CD3, 300446; CD45, 304044; CD8A, 301031, BioLegend). Stained cells were then washed twice with FACS buffer and the results acquired using NovoCyte (ACEA Biosciences). FlowJo (version 10) software (BD) was used to analyze flow cytometric data.

### 2.3. IHC, Histopathological Scoring, and Digital Image Analysis

Formalin-fixed, paraffin-embedded (FFPE) tumor sections (average size 1.2 cm^2^, 4 μm thick) were deparaffinized and rehydrated before heat-induced epitope retrieval at 110 °C for 5 min in a Decloaking NxGen Chamber TM (BioCare Medical, San Francisco, CA, USA). The unmasking buffer was selected according to the antibody product sheet recommendations with a preference for pH 6 (S2369, DAKO, Santa Clara, CA, USA) when more than one buffer was listed. Sections were allowed to cool down for 30 min, equilibrated in TBS-Tween 20 (0.1%), and endogenous peroxidase activity was quenched by 3% H_2_O_2_ for 10 min if horseradish peroxidase-linked reagents were to be used. Serum block was performed before applying the primary antibodies directed against CD11c (NCL-TL-TCD11c-T563, Novocastra, 1:75), CD8 (M7103, DAKO, 1:75), and Foxp3 (98377, Cell Signaling, 1:150). Secondary detection reagents were chosen considering the species origin of the primary antibody, and the type of enzyme label/visualization method preferred. For IHC, either ImmPress reagent anti-mouse IgG, peroxidase (MP-7402, Vector laboratories, Burlingame, CA, USA) or ImmPress reagent anti-mouse IgG, alkaline phosphatase (MP-5402, Vector laboratories, Burlingame, CA, USA) was used. For detection of Foxp3, the recommended SignalStain Boost reagent was used (8114S, Cell Signaling, Danvers, MA, USA). Chromogenic substrates were DAB peroxidase substrate (SK-4100, Vector laboratories) or Liquid permanent red (K0640, DAKO, Santa Clara, CA, USA). Protocols for double labeling were optimized for sequential IHC with a second heat induced epitope retrieval step at 80 °C for 5 min followed by 20 min cool down. Cell nuclei were counterstained with Mayer’s Hematoxylin (01820, Histolab, Stockholm, Sweden) before dehydration and mounting in permanent VectaMount mounting medium (H5000, Vector laboratories, Burlingame, CA, USA).

Histopathological scoring was performed blinded to patient outcome with a categorical scoring system (absent/present) and cell–cell interactions were scored as “no interactions” or “interactions”. The visual assessment was based on whole tissue section analysis and not hotspot analysis. A consultant pathologist (F.H.) scored presence of TLSs and intratumoral interactions in the liposarcoma subgroup, and a kappa value of 0.83 (very good agreement) was obtained between rater 1 (Y.S) and rater 2 (F.H.).

For TLS-based quantifications, tumor sections from four patients with mature TA-TLS reviewed by a pathologist (F.H.) were included. The sections were scanned with a Hamamatsu NanoZoomer 2.0-HT digital slide scanner at 400× magnification and visualized using the Nano Zoomer Digital Pathology (NDP) viewer software (U12388-01; NDP.view2 Viewing). Quantifications were performed with the QuPath open source software (https://github.com/petebankhead/qupath; accessed date 8 March 2021) [39], according to the localization: inside TLS (intra-TLS), close surrounding the TLS (peri-TLS) and far outside the TLS of interest (extra-TLS). Image type was set as (‘BRIGHTFIELD_OTHER’), and color deconvolution stains were set as (‘{“Name”: “H-DAB red”, “Stain 1”: “Hematoxylin”, “Values 1”: “0.56756 0.74313 0.35444”, “Stain 2”: “DAB”, “Values 2”: “0.26896 0.56792 0.7779”, “Stain 3”: “RED”, “Values 3”: “0.26395 0.81686 0.51291”, “Background”: “221 222 224”}’).

With the TLS as the center, the peri-TLS region was defined as a surrounding region outside the TLS, within a distance of 150 µm outside the border of the TLS; and the region outside the TLS was defined as an outer region of the peri-TLS, within a distance of 150 µm outside the border of the peri-TLS. Images were standardized to a common set of default DAB staining vector parameters. Cell detection script: (‘qupath.imagej.detect.cells.WatershedCellDetection’, ‘{“detectionImageBrightfield”: “Hematoxylin OD”, “requestedPixelSizeMicrons”: 0.5, “backgroundRadiusMicrons”: 8.0, “medianRadiusMicrons”: 0.0, “sigmaMicrons”: 1.5, “minAreaMicrons”: 10.0, “maxAreaMicrons”: 400.0, “threshold”: 0.1, “maxBackground”: 2.0, “watershedPostProcess”: true, “excludeDAB”: false, “cellExpansionMicrons”: 2.0, “includeNuclei”: true, “smoothBoundaries”: true, “makeMeasurements”: true}’).

For the classification of CD11c–CD8 interactions in TLS regions, one middle-sized TLS per patient was included in the analysis. The number of CD11c–CD8 interactions was defined as the number of CD8+ cells in direct contact with CD11c+ cells. An object classifier was set up based on the artificial neural network (ANN_MLP) algorithm of the software and was trained in half of the images in order to reach an optimal setting. Subsequently, the object classifier was applied for all images.

For the classification of Foxp3+ positive cells, three middle-sized TLSs per patient were included in the analysis. The single measurement classifier was set up with the following parameters: object filter: cell; channel filer: DAB; measurement: Nucleus: DAB OD mean. The thresholds were set and adjusted in order to get the optimized single classification.

The statistical comparisons of the percentage of CD11c–CD8 interactions and Foxp3+ cells in different regions were performed using one-way ANOVA with a two-sided alpha of 0.05. A *p*-value < 0.05 was considered as statistically significant. Statistical analysis was performed using the SPSS 24 software (IBM Corp, Chicago, IL, USA).

### 2.4. Opal^TM^ Multiplexing and Digital Image Analysis

Potential coexpression of proteins in the same cellular compartment was investigated with Opal^TM^ Multiplexing reagents (NEL811001KT, Akoya Biosciences, Marlborough, MA, USA). Tumor sections were prepared as described in 2.3. Primary antibodies against CD8 (M7103, DAKO, 1:100) and PD-1 (86163, Cell Signaling, 1:75) were incubated for 1 h and overnight, respectively. Secondary antibodies (Opal Polymer anti-mouse and rabbit HRP, ARH1001EA, Akoya Biosciences) were applied for incubation for 10 min at room temperature, followed by a 2-min wash in TBS-T 3 times. The Opal 520 fluorophore was used for CD8 detection and the Opal 620 fluorophore for PD-1 detection. Both Opal fluorophores were diluted 1:100 in 1X plus amplification diluent (FP1498, Akoya Biosciences) and incubated on the sections for 10 min at room temperature avoiding light. A heat-induced epitope retrieval with a pH 6 solution (S2369, DAKO) for 20 min at 95 °C was performed between the antibodies, and afterwards, before mounting with ProLong^TM^ Glass Antifade Mountant with NucBlue^TM^ stain (P36985, ThermoFisher, Waltham, MA, USA). The corresponding protocol was used for double immunofluorescence detecting CD11c (NCL-TL-TCD11c-T563, Novocastra, 1:75) together with either CD8 (as above), CD68 (M0876, DAKO, 1:60, Opal 520 fluorophore), or CD163 (NCL-L-CD163, Novocastra, 1:200, Opal 520 fluorophore). For detection of CD11c in combination with CD68 or CD163, the Opal 650 fluorophore was used.

Imaging was performed with an AxioObserverZ1 microscope system with LED illumination at 200× magnification. For the CD8/PD1 coexpression analysis, 3 × 3 tiled/stitched images from 3 different tumor areas of each patient were captured by optical sectioning (ApoTome). In total, 12 patients with CD11c-CD8 interactions were included for the CD8/PD1 multiplexing and subsequent digital image analysis. The Qupath software (see above) was used for detection of CD8 and PD1 immunopositive cells with NucBlue for nuclear detection. Cell detection for images was performed with default settings: Threshold was set to 100, requested pixel size to 0.227 μm, background radius to 8 μm, median filter to 0 μm, minimum area to 10,000 μm^2^, maximum area to 400,000 μm^2^, and cell expansion to 2 μm. Subsequently, single and double positive cells were detected with the artificial neural network (ANN_MLP) algorithm of the software by using the same settings in 7 images. Then via 7 images, the machine learning system was trained to detect single and double positive cells in the rest of the images. The total number of double positive cells (pseudo green and pseudo red) varied between 15 and 2607 (average 960) for each tiled/stitched image depending on cell density and marker expression. The results were exported automatically into a “.txt” file for further statistical analysis in Microsoft Excel 2019.

The same cell detection method was used for the CD11c+CD68+ and CD11c+CD163+ analyses. For the classification of CD11c, CD68, and CD163 positive cells, the single measurement classifier was set up with the following parameters: object filter: cell; channel filer: AF488 and AF594. The thresholds were set and adjusted in order to get the optimized single classification. Subsequently, the composite classifier was created and used for the cell counts.

### 2.5. Statistical Analysis and Transcriptomics

For the Karolinska STS cohort, statistical analysis of histopathological scoring was carried out using SPSS version 20.0 or 22.0 (IBM Corp., Chicago, IL, USA). Non-normal distribution was assumed, and all tests were double sided. Immune cell infiltrate scores were analyzed with categorical variable distribution using the Fisher’s exact test and the Mann–Whitney U comparison was used to compare numerical variables between groups. Bivariate analysis of covariance was done with Spearman’s ranked correlation. Overall survival (OS) was computed from the date of diagnosis to the date of last follow-up or death, and metastasis-free survival (MFS) from the date of diagnosis to the date of last follow-up or first distant metastasis. Survival analysis was carried out as per Kaplan–Meier, and the presence or absence of CD8+ cells, presence or absence of CD11c+ cells, and presence or absence of CD11c–CD8 cellular interactions were tested as dichotomous variables, with the log-rank test used to compare differences between groups. Prognostic factors were identified in univariable survival analysis and hazard ratios were calculated using multivariable cox-regression analysis (proportional hazards model) where size and age were analyzed as continuous parameters. A *p* value of <0.05 was considered significant.

For transcriptomic analyses, mRNA expression values for *ITGAX*, *CD8A*, *FOXP3*, *PDCD1,* and *PD-L1*/*CD274* were extracted from the TCGA sarcoma dataset with each gene subsequently divided into two groups of low and high expression based on a median cutoff. Contrast groupings for *ITGAX* and *CD8A* were constructed on the basis of this binary split as follows: Group 1: *ITGAX*^low^/*CD8A*^low^, Group 2: *ITGAX*^high^/*CD8A*^low^, Group 3: *ITGAX*^low^/*CD8A*^high^, and Group 4: *ITGAX*^high^/*CD8A*^high^. Additional contrast groupings were constructed according to the same principle. Kaplan–Meier analyses for individual genes and contrast groups were performed using the survival and survplot R packages with OS as the clinical endpoint. Statistical comparison of survival curves was performed using a log-rank test. Analyses were performed using the R statistical software.

For additional information about the statistical analyses performed, see description in each methods section and figure legend.

## 3. Results

### 3.1. Spatial Cross Presentation in Human STS Is Prognostic for Patient Survival

**Figure 1:**
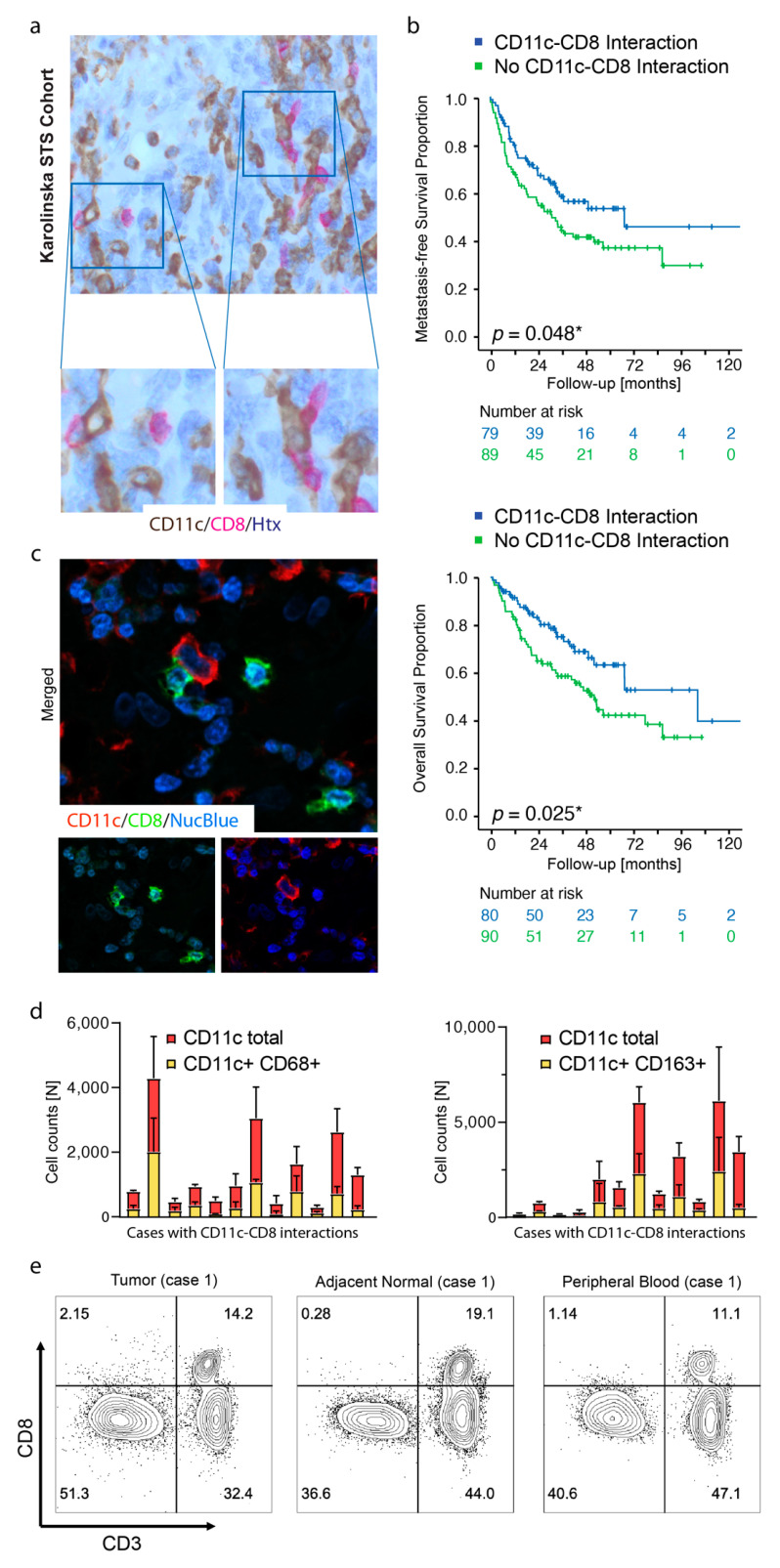
Spatial cross presentation in human STS is prognostic for patient survival. (**a**) Microphotograph with double immunohistochemistry (IHC) visualizing direct cell–cell interactions between CD11c+ cells and CD8+ cells; (**b**) Kaplan–Meier survival analysis using the log-rank test for comparison between absence or presence of CD11c–CD8 cellular interactions detected by double IHC in tumors from the Karolinska STS cohort. * *p* < 0.05; *p* < 0.05 is considered significant; (**c**) Double IF (Opal multiplexing) visualizing representative single-positive CD11c+ cells and single-positive CD8+ cells. Blue pseudocolor = nuclei; (**d**) QuPath digital image quantification of CD11c+ cells with or without coexpression of the macrophage markers CD68 (left) and CD163 (right). Each bar represents an individual case displaying CD11-CD8 interactions; (**e**) Flow cytometry for CD8 and CD3 in a preparation of freshly isolated CD45+ cells from a 76-year-old male with UPS, G3 displaying intratumoral CD11c–CD8 interactions in sections from the same tumor.

CD11c and CD8 expression was initially assessed by IHC of whole tissue sections from the Karolinska STS cohort (Figure 1a). Clinicopathological correlations indicated that neither CD11c nor CD8 expression was associated with patient MFS or OS (CD11c, MFS log-rank *p* = 0.912, OS log-rank *p* = 0.371; CD8, MFS log-rank *p* = 0.770, OS log-rank *p* = 0.438). However, presence of direct cell–cell interactions between CD11c+ cells and CD8+ cells correlated with superior MFS and OS (Figure 1b). These results were in line with the hypothesis that a certain spatial distribution and/or cell density of APCs and CD8+ T cells reflects a favorable tumor immune microenvironment.

CD11c is assumed to be expressed not only by conventional dendritic cells, but also pro-inflammatory macrophages with the ability to cross present dead cell-associated antigens to CD8+ T cells in tumors [30]. To further characterize CD11c+ cells in STS, a subset of tumors with CD11c–CD8 interactions, was next analyzed by immunostaining with Opal^TM^ multiplexing reagents. Firstly, double immunofluorescence confirmed that CD11c and CD8 were typically not expressed by the same cell population (Figure 1c). Secondly, a subset of CD11c+ cells was identified as macrophages, either with the CD68 pan-macrophage marker or the tumor-associated macrophage marker CD163 (Figure 1d, Figure S1a,b). Characterization of CD8+ cells was done by flow cytometry, where 87% of the CD8+ cells were positive for CD3 in one of the tumors with CD11c–CD8 interactions (Figure 1e). Flow cytometry from two additional cases confirmed low abundance of intratumoral CD8+CD3- cells, and the CD8+ cells were hereby assigned T cell identity (Figure S2).

Correlations with survival were further tested in a multivariable cox-regression analysis with other established prognostic factors such as size and grade, adjusting for age and sex. In this analysis, CD11c–CD8 interactions remained statistically significant for improved MFS and OS (Table 2). A limitation of the study was the heterogeneity of the Karolinska cohort (e.g., different subtypes with different grades). Still, the results provide support for CD11c–CD8 interactions as an independent biomarker with prognostic information beyond existing biomarkers.

### 3.2. TLSs Are Found in Smaller Tumors and Display High Density of CD11c+ Cells and CD8+ Cells

**Figure 2:**
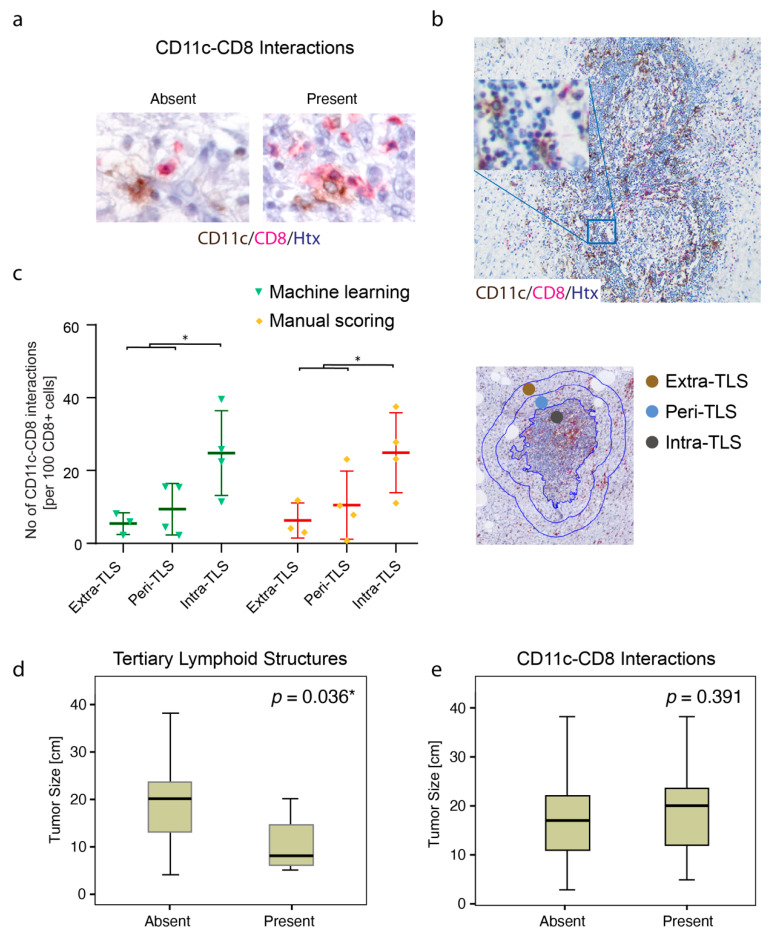
Tertiary lymphoid structures (TLSs) are found in smaller tumors and display high density of CD11c+ cells and CD8+ cells. (**a**) Microphotographs with double IHC visualizing absence or presence of intratumoral cell–cell interactions between CD11c+ and CD8+ cells in liposarcoma; (**b**) Microphotograph with a mature intratumoral TLS displaying high density of CD11c+ cells and CD8+ cells; (**c**) Quantification of CD11c–CD8 interactions/100 CD8+ cells outside (extra-TLS and peri-TLS) or inside (intra-TLS) tumor-associated tertiary lymphoid structures (TA-TLS) formations (+/−SD). Each dot in the graph represents either manual scoring or the QuPath image quantification result originating from one out of four cases. Statistical comparisons were performed with One-way Anova comparing intra-TLS regions with regions outside the TLS (close and distant). * *p* < 0.05; *p* < 0.05 is considered significant; (**d**) Box-plot visualization of presence of TLSs in relation to tumor size (Mann–Whitney U test); (**e**) Box-plot visualization of presence of CD11c–CD8 interactions in relation to tumor size (Mann–Whitney U test). * *p* < 0.05; *p* < 0.05 is considered significant.

Intratumoral cell–cell interactions between CD11c+ APCs and CD8+ T cells were explored more in detail in liposarcoma (Figure 2a). Multiple CD11c–CD8 interactions were particularly found in tumors with TA-TLSs (Figure 2b). Furthermore, it was confirmed that CD11c–CD8 interactions could be scored either by manual counting or by digital image analysis independent of the location to the TA-TLS (Figure 2c). Smaller tumors more often displayed mature TA-TLSs with germinal centers (Figure 2d), and tumors with TA-TLSs were always categorized as positive for the interaction. However, the analysis demonstrated that there were only five patients with TA-TLSs. This indicated that presence of mature TA-TLSs is a less frequent phenomenon compared to presence of CD11c–CD8 interactions. Notably, the presence of CD11c–CD8 interactions did not seem to be affected by tumor size (Figure 2e).

### 3.3. Foxp3+ Cells Reside in TA-TLSs

**Figure 3:**
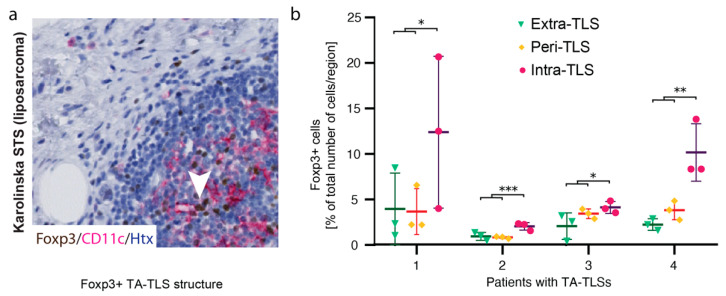
Foxp3+ cells reside in TA-TLSs. (**a**) Representative image of CD11c+ cells and Foxp3+ cells residing primarily within a TA-TLS (intra-TLS) and not in the peri-TLS region. (**b**) Quantification of Foxp3+ cell numbers in relation to total cell numbers and TA-TLS formation (+/−SD). Each dot in the graph represents the QuPath image quantification result originating from one out of three middle-sized TA-TLSs. Statistical comparisons were performed with One-way Anova comparing intra-TLS regions with regions outside the TLS (close and distant). * *p* < 0.05, ** *p* < 0.01, *** *p* < 0.001; *p* < 0.05 is considered significant.

Regulatory T cells in TA-TLSs have been shown experimentally to suppress anti-tumor T cell responses [24]. To explore whether regulatory T cells could potentially interfere with CD11c+ APC function, presence and distribution of Foxp3+ cells were next explored and quantified in detail in four liposarcoma cases. From digitally scanned whole tissue sections, regions of interest were selected starting from the center of each TLS (Figure S3a). Subsequent image analysis revealed that Foxp3+ cells particularly resided in the intra-TLS regions (Figure 3a,b), but could also be sparsely distributed in tumors without mature immune cell clusters (Figure S3b). This observation was consistent with the notion that Foxp3+ T regulatory cells are present where there is a need for an immunological brake.

### 3.4. ITGAX Expression Correlates with HLA Genes, Whereas CD8A Correlates with CD3 Chains and CD8B

To further investigate the function of CD11c+ cells in STS, the cbioportal online tool was used for explorative Spearman correlations in the publicly available TCGA sarcoma cohort [40,41]. The results demonstrated strong correlations between expression of the *ITGAX* gene, encoding for the CD11c protein, and multiple classical MHC class II molecules, such as *HLA-DRA*, *HLA-DPB1,* and *HLA-DPA1* (Table S1). *ITGAX* further correlated with, e.g., the costimulatory molecule *CD86* (correlation coefficient 0.72), the immune cell activation and differentiation marker *CD48* (correlation coefficient 0.77), and *CD5* (correlation coefficient 0.68), which is a receptor suggested to be involved in T cell survival and proliferation. Other examples of significant correlations were MHC class I molecules such as HLA-A (correlation coefficient 0.59), HLA-B (correlation coefficient 0.56), and HLA-C (correlation coefficient 0.56), even though these were weaker compared to those with MHC class II molecules. These findings were in line with the notion that CD11c+ cells are involved in antigen presentation, as well as T cell activity, in STS.

T cell function and/or identity of CD8A-expressing cells were similarly investigated by Spearman correlation in the same cohort (Table S2). The top correlated gene was CD3E (correlation coefficient 0.908), and strong correlations were also noted for the other CD3 chains involved in T cell receptor signaling (CD3D, correlation coefficient 0.889; CD3G, correlation coefficient 0.884). Moreover, presence of functional CD8α-CD8β heterodimers in the tumors was supported by a strong correlation with the CD8B gene (correlation coefficient 0.871).

### 3.5. High Gene Expression Levels of ITGAX Together with CD8A Are Prognostic by Transcriptomics, Irrespective of FOXP3 TME Levels, in Human STS

**Figure 4:**
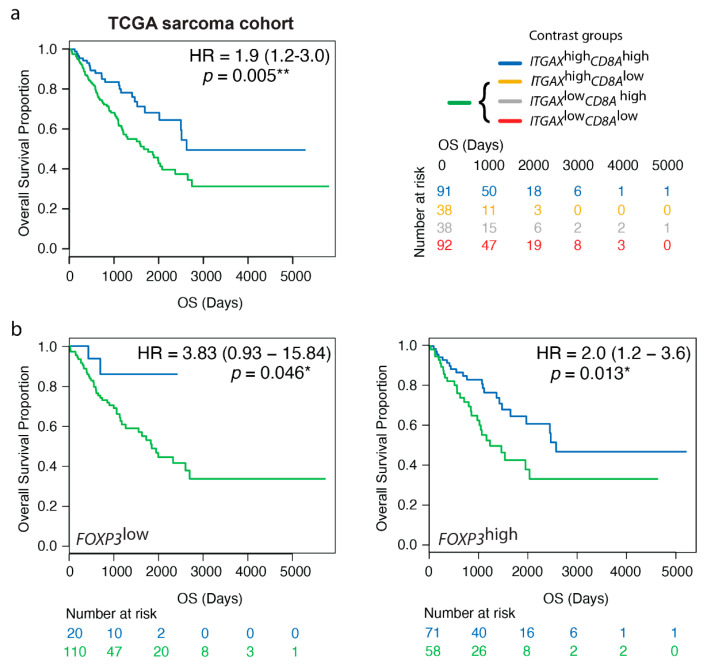
High gene expression levels of *ITGAX* together with *CD8A* are prognostic by transcriptomics, irrespective of *FOXP3* levels, in human STS. (**a**) Contrast group analysis of gene expression levels of *ITGAX* (CD11c) and *CD8A* (CD8) in the TCGA sarcoma cohort; (**b**) Contrast group analysis of gene expression levels of *ITGAX* (CD11c) and *CD8A* (CD8) in the TCGA sarcoma cohort where *FOXP3*^low^ and *FOXP3*^high^ indicate subgroupings according to total *FOXP3* levels in the TME. * *p* < 0.05, ** *p* < 0.01; *p* < 0.05 is considered significant.

The hypothesis that a certain spatial distribution and/or cell density of APCs and CD8+ T cells reflects a favorable tumor immune microenvironment was further investigated by an independent methodology in the TCGA sarcoma cohort, which had a similar STS subtype composition as the Karolinska STS cohort (see Material and Methods). All tumor samples were placed in one out of four contrast groups according to standard procedures for contrast group analysis with binary median split for both genes (Figure S4a). Also, this approach supported a prognostic value of CD11c+ cells together with CD8+ cells in STS (Figure 4a). This was further confirmed in a separate subgroup analysis of leiomyosarcoma only (Figure S4b). This subtype was represented by a comparably high number of cases in the TCGA sarcoma cohort, but not in the Karolinska STS cohort where the prognostic role of CD11c+ cells together with CD8+ cells was initially detected. Importantly, the *ITGAX*^high^*CD8A*^high^ gene signature remained prognostic in leiomyosarcomas alone, which indicated a relevance of CD11c+ cells together with CD8+ cells also in leiomyosarcoma.

The whole TCGA sarcoma cohort was next explored regarding immune regulatory Foxp3+ cells. Importantly, when *FOXP3* levels were specifically considered, the *ITGAX*^high^*CD8A*^high^ gene signature remained prognostic in cases with a *FOXP3*^high^ TME (Figure 4b). This indicated that even if Foxp3+ cells may potentially dampen lymphocyte function, they do not completely abolish the associated anti-tumor activities in immunologically “hot” areas with CD8+ T cell nfiltration.

### 3.6. The Prognostic Value of the ITGAX^high^CD8A^high^ Gene Signature Is Maintained in a CD274 (PD-L1)-Rich TME

**Figure 5:**
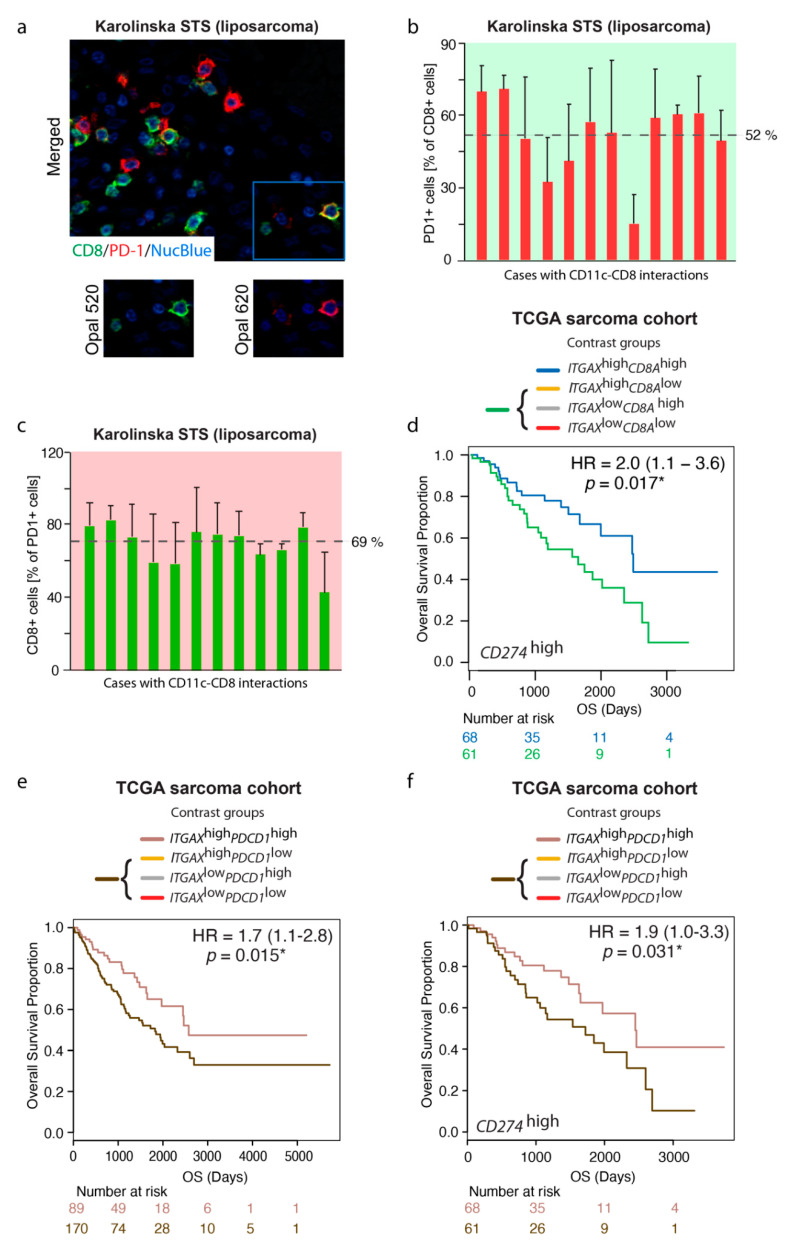
The prognostic value of the *ITGAX*^high^*CD8A*^high^ gene signature is maintained in a *CD274* (PD-L1)-rich TME. (**a**) Double IF (Opal™ multiplexing) visualizing three types of immunopositive cells: CD8+/PD1+, CD8+/PD1-, and CD8-/PD1+ in liposarcoma. Blue pseudocolor=nuclei; (**b**) QuPath image quantification of PD1+ cells among the CD8+ T cell population (+SD from 3 different 3 x 3 tiled/stitched images). The average percentage of positive cells is indicated with a dotted line. Each bar represents an individual case displaying CD11-CD8 interactions; (**c**) QuPath digital image quantification of CD8+ cells among the PD1+ cell population (+SD from 3 different 3 × 3 tiled/stitched images). The average percentage of positive cells is indicated with a dotted line. Each bar represents an individual case displaying CD11-CD8 interactions; (**d**) Contrast group analysis of gene expression levels of *ITGAX* (CD11c) and *CD8A* in a *CD274*^high^ TME in the TCGA sarcoma cohort comparing the *ITGAX*^high^*CD8A*^high^ group with the other three contrast groups combined (*ITGAX*^high^*CD8A*^low^*+ITGAX*^low^*CD8A*^high^*+ITGAX*^low^*CD8A*^low^); (**e**) Contrast group analysis of gene expression levels of *ITGAX* (CD11c) and *PDCD1* (PD1) in the TCGA sarcoma cohort comparing the *ITGAX*^high^*PDCD1*^high^ group with the other three contrast groups combined (*ITGAX*^high^*PDCD1*^low^*+ITGAX*^low^*PDCD1*^high^*+ITGAX*^low^*PDCD1*^low^); (**f**) Contrast group analysis of gene expression levels of *ITGAX* (CD11c) and *PDCD1* (PD1) in a *CD274*^high^ TME in the TCGA sarcoma cohort comparing the *ITGAX*^high^*PDCD1*^high^ group with the other three contrast groups combined (*ITGAX*^high^*PDCD1*^low^*+ITGAX*^low^*PDCD1*^high^*+ITGAX*^low^*PDCD1*^low^). * *p* < 0.05; *p* < 0.05 is considered significant.

Immunotherapy is a promising anti-cancer strategy in which the immune checkpoint protein PD1 is of interest. The PD1-encoding *PDCD1* gene was therefore selected as a candidate for further exploratory gene correlation analysis. In this analysis, the *CD8A* gene was identified as one of the top three genes correlating with *PDCD1* (correlation coefficient 0.827; q value 2.05 × 10^−60^, Benjamini–Hochberg FDR correction). To investigate cell type-specific localization in the TME, protein coexpression of CD8 and PD1 was subsequently studied by double immunofluorescence with the Opal^TM^ multiplexing technology in FFPE tumors (Figure 5a). Only liposarcomas were included, and only those that displayed CD11c–CD8 interactions. Despite this selection of tumors with similar characteristics, PD1 positivity among CD8+ cells clearly varied between patients, ranging from 14% to 71% (Figure 5b). Likewise, CD8 positivity varied among PD1+ cells, ranging from 43% to 82% (Figure 5c). This heterogeneity in coexpression indicated presence of different subpopulations of positive cells, of which some might be responsive to PD1-targeting therapy.

PD1, CTLA-4, and additional checkpoint molecules are commonly referred to as exhaustion markers. To investigate whether there were any signs of dampened CD8+ T cell activity mediated by PD-L1/PD1 signaling in tumors with high density of CD11c+ APCs, a contrast group analysis specifically addressed whether the *ITGAX*^high^*CD8A*^high^ gene signature in the TCGA sarcoma cohort was detected also in a *CD274* (encoding PD-L1)-rich TME. The results demonstrated that the prognostic value of *ITGAX*^high^*CD8A*^high^ was maintained, and thereby, seemingly not suppressed by PD-L1 (Figure 5d).

Given the strong correlation between *PDCD1* and *CD8A* in the TCGA sarcoma cohort, the next analysis addressed whether PD1+ cells harbored a similar prognostic potential as CD8+ cells in combination with CD11c+ cells. A contrast group analysis revealed that also the *ITGAX*^high^*PDCD1*^high^ signature correlated with favorable OS (Figure 5e). The analysis further demonstrated that the prognostic value was maintained in a *CD274*^high^ TME, which again implied that CD8+ T cell activity was not fully inhibited by the presence of PD-L1 in the TME (Figure 5f). Notably, it appeared that tumors without the *ITGAX*^high^*PDCD1*^high^ gene signature, but with high *CD274* levels, were associated with worse prognosis. Whether these patients would benefit from immunotherapy remains to be investigated.

## 4. Discussion

The clinical success of cancer immunotherapy in, e.g., melanoma and non-small cell lung cancer, nurtures further explorative studies on immune cell activation, maturation, and suppression in other malignancies. Sarcomas are in several aspects different from the so called immunologically “hot” tumors with prevalent lymphocyte infiltration. Investigated subtypes typically display a lower number of mutations and sometimes subtype-dependent expression of checkpoint molecules [12,42]. Still, early data suggests beneficial responses to anti-PD1 therapy in certain subtypes and patients [43]. The potential risk of disease hyperprogression following PD1 blockade should however be considered, and better knowledge about cellular crosstalk from a prognostic and/or response predictive perspective in the TME is needed [44,45,46].

This study aimed to characterize the prognostic value of CD11c-expressing APCs together with CD8+ T cells in different molecular TMEs considering pathways with potential immunosuppressive activities. Spatial cross presentation was specifically introduced as a novel concept where CD11c–CD8 cellular interactions in FFPE tumor tissue was associated with improved MFS and OS. CD11c is typically expressed by professional APCs, including dendritic cells and macrophages. CD8 is a co-receptor for the T cell receptor on cytotoxic T cells, but a subpopulation of CD8+ cells could also represent uncommon subsets of other immune cells such as myeloid cells and gamma-delta T cells. Our study does not rule out that a minority of other cell types expressing either CD11c or CD8 are present in STS, but the initial coexpression analysis by immunostaining, flow cytometry, and transcriptomics suggested that dendritic cells, macrophages, and cytotoxic T cells were the most dominating cell types associated with CD11c and CD8 expression.

Notably, direct cell–cell interactions were easily scored with regular light microscopy, and intratumoral areas were scored separately from peritumoral areas. One observation from the liposarcoma subgroup analysis was that CD11c–CD8 interactions were always found in tumors with TLSs. Evidence from several malignancies indicated beneficial immunogenic activities and response to immunotherapy linked to presence of TA-TLSs [22]. The heterogeneous characteristics of mesenchymal tumors must, however, be considered before directly translating these findings to STS in general [21,47]. Our study indicated that TA-TLSs were more frequently found in smaller tumors, but the clinical significance of this observation must be evaluated. The analysis further demonstrated that scoring for CD11c–CD8 cellular interactions, instead of presence of TA-TLSs, allowed for inclusion of more patients in the superior prognosis group.

We also noted that Foxp3+ cells resided inside and in the margins of mature TA-TLSs. Regulatory T cells expressing Foxp3 are often considered to dampen anti-tumoral activities, but their function in STS remains unclear. Although a balanced activity in general might be beneficial for the patient, it is too early to say whether Foxp3+ cells substantially regulate anti-tumor responses based on their spatial presence and accumulation in TA-TLSs. To begin with, it must be considered that TA-TLSs are almost by definition associated with a more favorable prognosis in solid tumors. Upcoming studies should therefore preferably address this question in more sophisticated model systems specifically designed for this purpose. Our contrast group analysis, however, clearly demonstrated that overall *FOXP3* levels did not significantly alter the prognostic value of the *ITGAX*^high^*CD8A*^high^ gene signature.

Similarly, the prognostic value of *ITGAX*^high^*CD8A*^high^ was also maintained in a TME with high *CD274* (PD-L1) levels. Typically, presence of the PD-L1 protein and its receptor PD1 together with CTLA-4 and other inhibitory receptors could indicate lymphocyte dysfunction. However, double immunofluorescence for CD8 and PD1 in tumors with CD11c–CD8 interactions rather indicated a notable heterogeneity among CD8+ T cells, which could reflect different degrees of functional activation. This was also supported by transcriptomics; without favoring the usage of *PDCD1* over *CD8A*, the *ITGAX*^high^*PDCD1*^high^ gene signature was likewise significant in a contrast group analysis. The prognostic signature was consistent in a *CD274*-rich TME, which again suggested presence of at least a proportion of cells that are not fully suppressed by PD-L1/PD1 signaling in tumors with high density of CD11c+ cells and CD8+ cells.

Our analysis of the Karolinska STS cohort mainly focused on liposarcoma and UPS, but some of the findings were also validated by transcriptomics in the TCGA sarcoma cohort dominated by leiomyosarcoma, liposarcoma, and UPS. More in depth studies are currently needed to understand the potential role of PD-L1/PD1 expression in distinct STS subtypes, and to what extent TA-TLSs are essential for immune cell function related to CD11c+ APCs in direct contact with CD8+ T cells. The role of other PD1+ lymphocytes in this setting would also be interesting to learn more about. It is for instance expected that CD4+ T cells are required to license dendritic cells prior to effective CD8+ T cell priming [48]. At present, it is also unclear whether the spatial interaction between CD11c+ APCs and CD8+ T cells involves effective MHC class I-mediated T cell priming at the molecular and functional level [49]. Nevertheless, our findings reveal a novel molecular biomarker in STS, and furthermore, provide a rationale for continued studies on functional interactions between APCs and T cells in the STS microenvironment.

## 5. Conclusions

There is currently a great need for novel functional biomarkers for immune-monitoring of patients. The present study has identified clinically relevant alterations in the tumor immune microenvironment, where intratumoral cell–cell interactions between marker-defined CD11c+ APCs and CD8+ T cells emerged as a novel prognostic marker associated with superior patient survival in STS. Notably, direct cell–cell interactions can be scored on an individual case basis and with regular light microscopy.

## Figures and Tables

**Table 1:** Characteristics of the Karolinska soft tissue sarcoma (STS) cohort (inclusion years 2011–2020).

Cohort Characteristics	Undifferentiated Pleomorphic Sarcoma (UPS)	Liposarcoma	Other ^1^
**Patients**	115	48	14
**Age (years)**			
Mean/Median	70/71	68/68	68/68
Range (min–max)	20–94	26–86	24–89
**Sex (N)**			
Male	53	29	8
Female	62	19	6
**Grade (FNCLCC)**			
G1		9	1
G2	27	14	5
G3	88	25	6
GX/n.d.			2
**Primary tumor size (cm)**			
Mean/Median	9/8	18/19	9/7
Range (min–max)	1–35	3–38	2–28
**Location**			
Superficial	48		5
Deep	65	48	9
Missing/n.d.	2		
**Resection margin**			
Intralesional	19	21 * (42 **)	3
Marginal	49	24 * (6 **)	7
Wide	44	3 * (0 **)	4
Radical (amputation)	1		
Missing/n.d.	2		
**Growth pattern**			
Infiltrative	88	48	10
Pushing border	25		2
Missing/n.d.	2		2
**Adjuvant therapy**			
Chemotherapy	13	2	2
Radiation	14	4	9
**Vascular involvement**			
Present	14	1	3
Absent	98	47	11
Missing/n.d.	3		
**Overall survival (OS) (%)**			
12 months	84	92	93
36 months	58	82	79
60 months	45	66	56
**Metastasis-free survival (MFS) (%)**			
12 months	71	81	79
36 months	54	42	64
60 months	47	37	54

^1^ Myxofibrosarcoma (6), angiosarcoma (2), malignant peripheral nerve sheath tumor (2), synovial sarcoma (2), malignant solitary fibrous tumor (1), leiomyosarcoma (1). n./d. = not determined, * dedifferentiated component, ** well-differentiated component.

**Table 2:** Multivariable analysis (cox-regression) of clinicopathological features and survival in the Karolinska STS cohort.

Clinicopathological Parameter	MFSHR (95% C.I.)	P (Cox)	OSHR (95% C.I.)	P (Cox)
Age (continuous, years)	1.016	0.072	1.022	0.027 *
	(0.999–1.034)		(1.002–1.042)	
Sex (male)	1.057	0.807	1.221	0.415
	(0.678–1.648)		(0.755–1.974)	
Grade, FNCLCC (G3)	2.412	0.001 **	3.339	0.000 ***
	(1.426–4.081)		(1.783–6.255)	
Size (cm, continuous)	1.042	0.001 **	1.029	0.040 *
	(1.017–1.067)		(1.001–1.057)	
CD11c–CD8 interactions	0.561	0.012 *	0.479	0.004 **
(present)	(0.357–0.881)		(0.290–0.792)	

HR = hazard ratio, C.I. = confidence interval, * *p* < 0.05; ** *p* < 0.01; *** *p* < 0.001; *p* < 0.05 is considered significant.

## Data Availability

Restrictions apply to the availability of the data from the Karolinska STS cohort due to ethical standards and data protection. RNA-sequencing data from the TCGA sarcoma cohort can be accessed from NIH genomic data commons (GDC) database (https://gdc.cancer.gov; accessed date 17 August 2020).

## Supplementary Materials

The following are available online at https://www.mdpi.com/2072-6694/13/5/1175/s1, Figure S1: A subpopulation of CD11c+ cells expresses macrophage markers. Figure S2: The majority of the isolated CD8+ cells from primary tumors express CD3. Figure S3: Digital image analysis of Foxp3+ cells in relation to presence of TA-TLSs. Figure S4: High gene expression levels of *ITGAX* together with *CD8A* are prognostic by transcriptomics in human STS. Table S1: *ITGAX* gene correlations. Table S2: *CD8A* gene correlations.
