# Supplementary material for: CD11c-CD8 Spatial Cross Presentation: A Novel Approach to Link Immune Surveillance and Patient Survival in Soft Tissue Sarcoma
Source: Cancers (Basel). 2021 Mar 9;13(5):1175. doi: 10.3390/cancers13051175 (PMC7967210; doi:10.3390/cancers13051175)
Supplement: Supplementary file 1 [file cancers-13-01175-s001.zip › Supplementary Figures.pdf]

cancers
MDPI

Supplementary Materials

CD11c-CD8 Spatial Cross Presentation: A Novel Approach to Link Immune Surveillance and Patient Survival in Soft Tissue Sarcoma

Yanhong Su, Panagiotis Tsagkozis, Andri Papakonstantinou, Nicholas P. Tobin, Okan Gultekin, Anna Malmerfelt, Katrine Ingelshed, Shi Yong Neo, Johanna Lundquist, Wiem Chaabane, Maya H. Nisancioglu, Lina W. Leiss, Arne Östman, Jonas Bergh, Saikiran Sedimbi, Kaisa Lehti, Andreas Lundqvist, Christina L. Stragliotto, Felix Haglund and Monika Ehnman

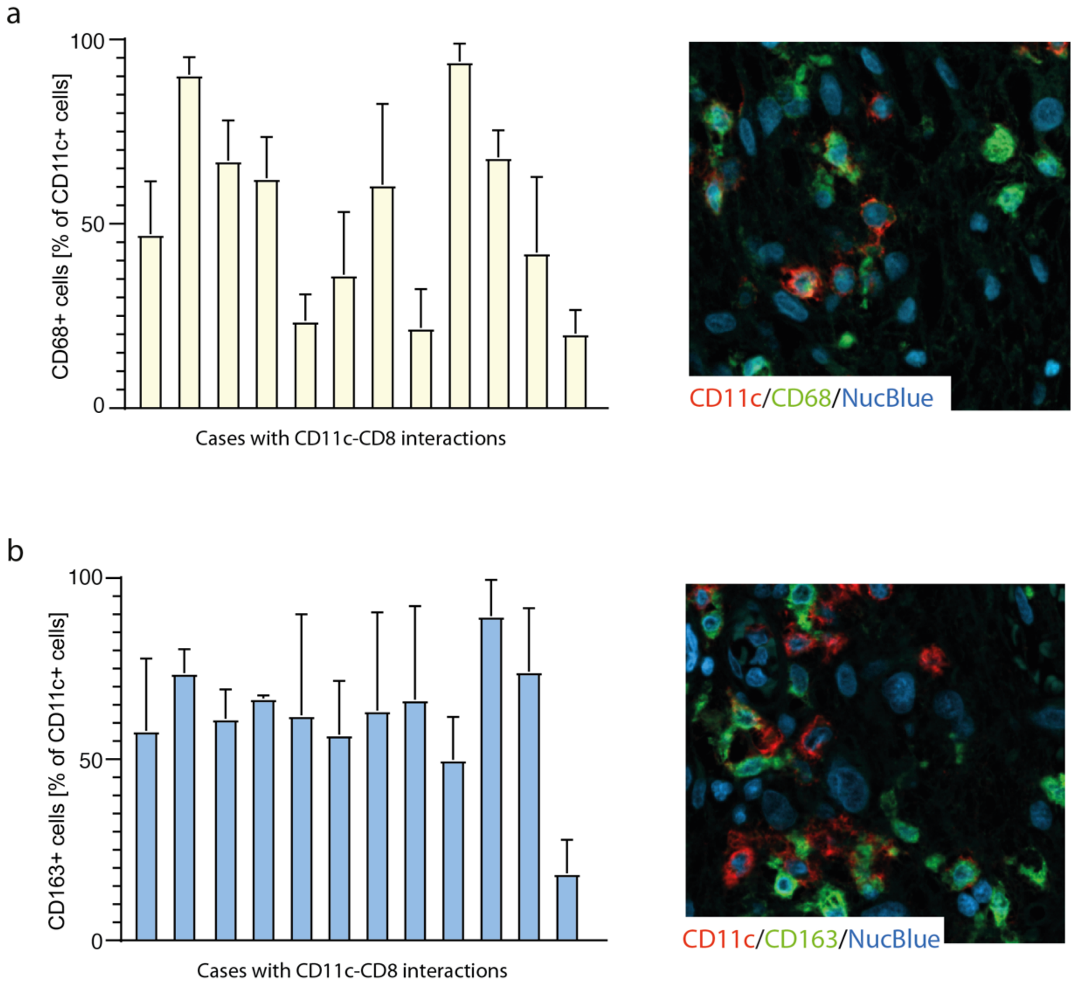

Supplementary Figure S1. A subpopulation of CD11c+ cells expresses macrophage markers. (a) QuPath digital image quantification of CD11c+ cells expressing the macrophage marker CD68 in STS cases with CD11c-CD8 interactions. Each bar represents an individual case displaying CD11-CD8 interactions; (b) QuPath digital image quantification of CD11c+ cells expressing the macrophage marker CD163 in STS cases with CD11c-CD8 interactions. Each bar represents an individual case displaying CD11-CD8 interactions.

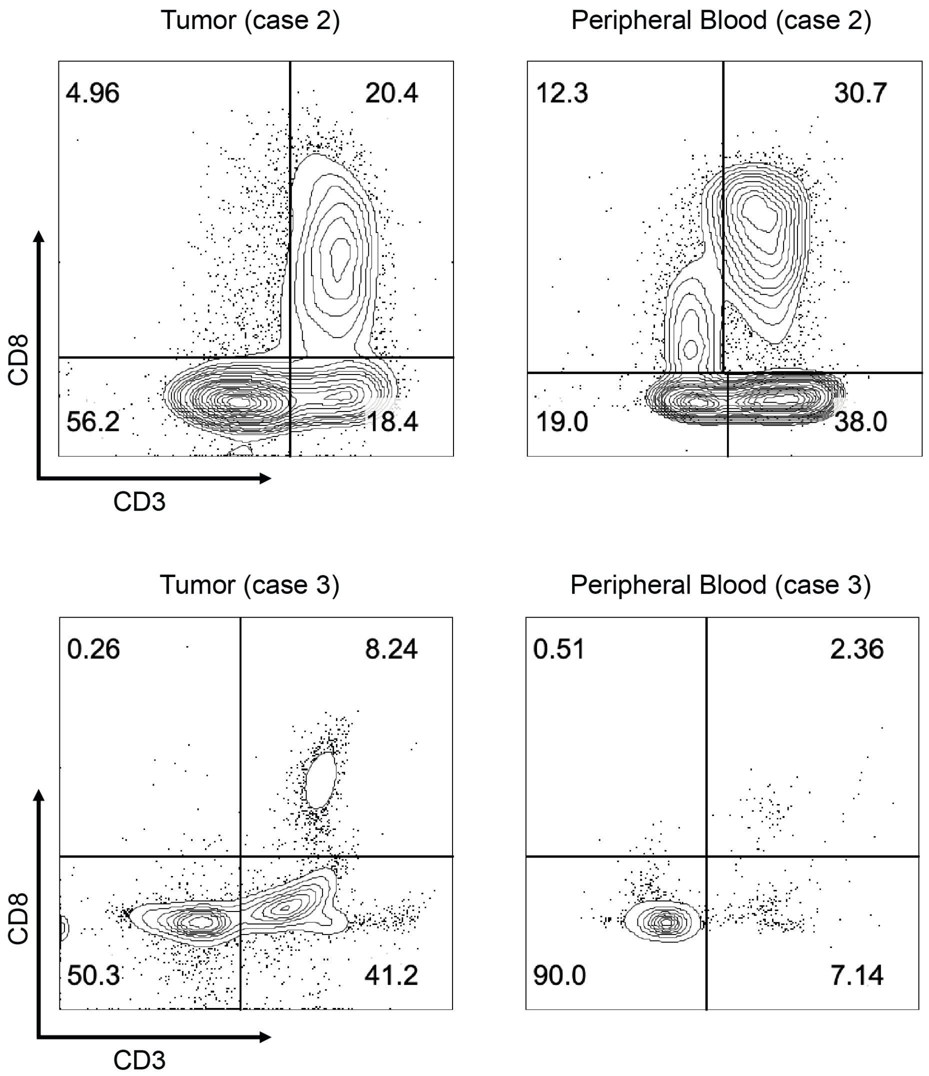

Supplementary Figure S2. The majority of the isolated CD8+ cells from primary tumors express CD3. Flow cytometry for CD8 and CD3 in preparations of freshly isolated CD45+ cells from two different patients. Case 2 is a dedifferentiated liposarcoma, G3 from a 56-year-old male (top), and case 3 is a UPS, G3 from a 59-year-old female (bottom).

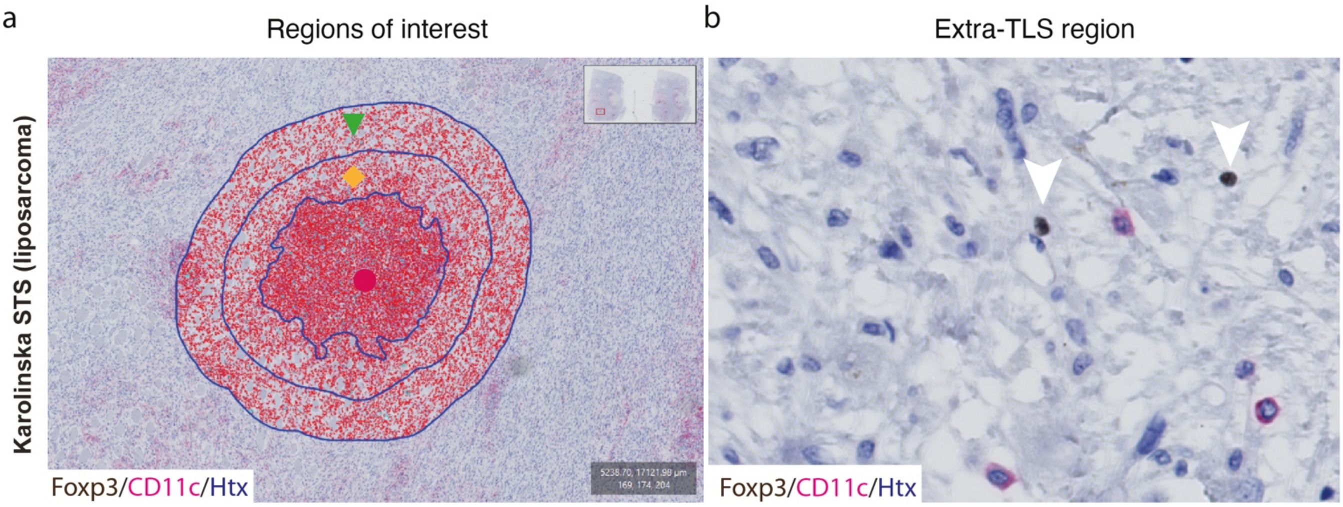

Supplementary Figure S3. Digital image analysis of Foxp3+ cells in relation to presence of TA-TLSs. (a) Representative example of how regions of interest were selected for digital image analysis of Foxp3+ cells residing primarily within a TA-TLS (intra-TLS, red label) and not in the peri-TLS region (yellow label) or in the extra-TLS region (green label) (b) Representative image of an extra-TLS region with sparsely distributed Foxp3+ cells.

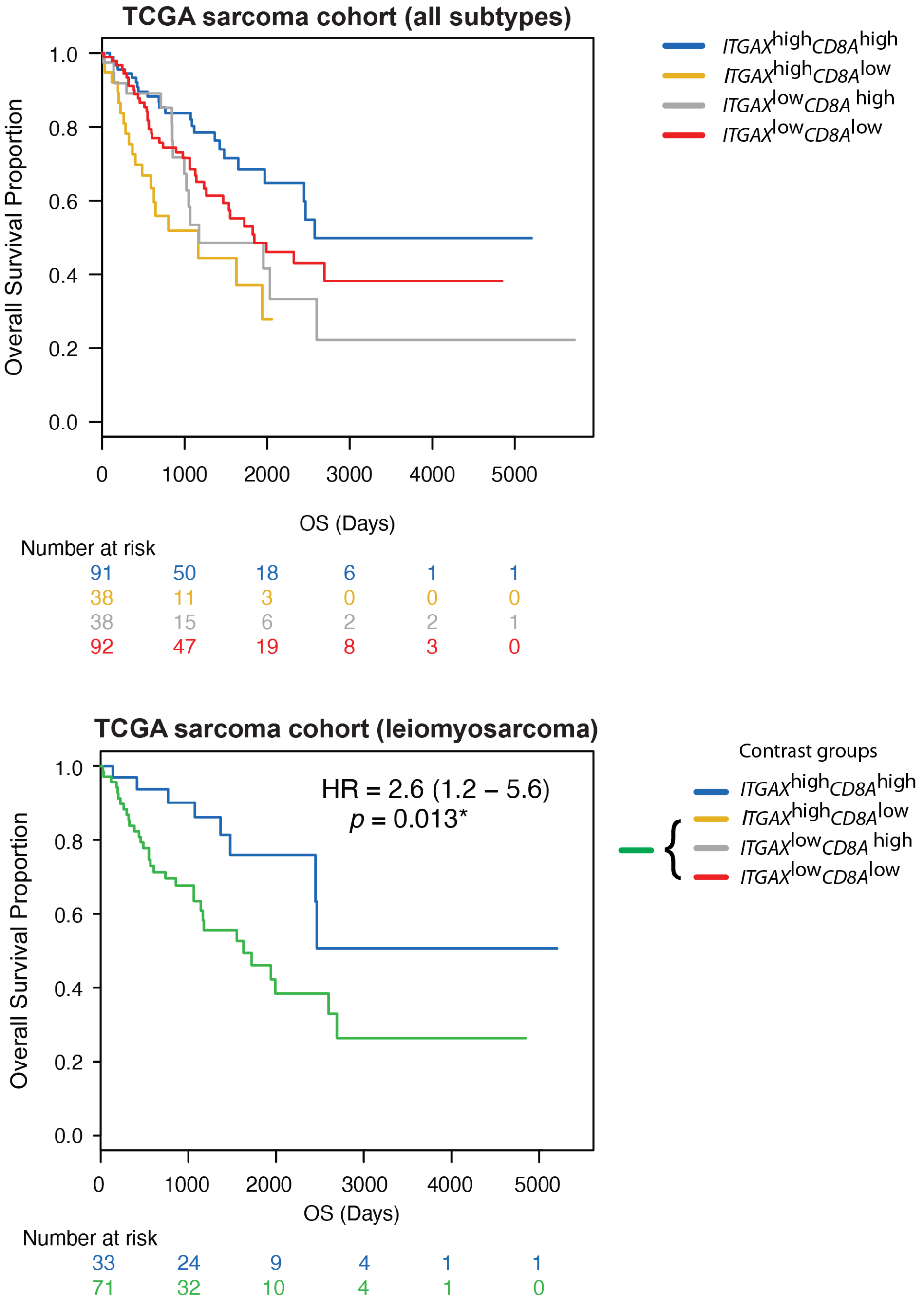

Supplementary Figure S4. *ITGAX-CD8A* expression levels are prognostic in STS. (a) Contrast group analysis of gene expression levels of *ITGAX* (CD11c) and *CD8A* (CD8) in the TCGA sarcoma cohort. (b) Contrast group analysis of gene expression levels of *ITGAX* (CD11c) and *CD8A* (CD8) in leiomyosarcoma subgroup analysis.
